# Multimodal Data Sharing, Analysis Workflows, and Biosample Discovery in a Global Consortium Approach: An Architectural Framework for Secure, Collaborative Data Analysis

**DOI:** 10.1002/alz70856_100504

**Published:** 2025-12-24

**Authors:** Vijay Sureshkumar

**Affiliations:** ^1^ Gates Ventures, Seattle, WA, USA

## Abstract

**Background:**

Biomedical research analysis often requires researchers to have **advanced programming capabilities** to query and harmonize datasets, build cohorts, and reproduce prior analyses in their own local environment. Extensive programming effort and familiarity with cloud services are required for executing workflows and pipelines across multiple datasets. **GRIP aims to address these barriers** for physicians and scientists alike.

**Method:**

GRIP is built with **open‐source technologies** capable of handling diverse workflows and research pipelines. It provides **ready‐to‐use tools** for researchers in medical imaging, ‘omics, digital health, and other data‐driven fields. The **cloud‐native architecture**, leveraging a Kubernetes (K8S) cluster, ensures scalability and compatibility with Open Container Initiative (OCI) standards. GRIP manages workflow templates, instances, and a registry of compute engines, enabling researchers to define, execute, and reproduce analyses across different datasets. To enhance reproducibility, GRIP tracks all relevant parameters, including dataset/tool versions and algorithm settings. This comprehensive orchestration and tracking capability ensures **consistent and transparent research results**. The GRIP Workflows architecture consists of several key components: **community‐built** Research Modules, Workflow Engine, Visualization Tools, and Infrastructure.

**Result:**

Progress to date includes multiple GRIP components, including data collection and curation (a mobile app for speech and cognitive test data collection), workflows and pipeline (imaging, speech, and ‘omics workflows on Azure Trusted Research Environment), data sharing (A4/LEARN clinical trials data sharing ‐ **white label implementation**), and visualization (web‐based FreeBrowse for FreeSurfer output visualization & OHIF Web Based Image Viewers).

**Conclusion:**

GRIP's initial platform launch, scheduled for release at the end of Q1 2025, aims to support dementia and aging‐focused researchers wishing to analyze imaging, digital, and/or ‘omics data in a secure, collaborative cloud environment. GRIP's offerings will continue to expand from the initial collection of modular tools built by our AD research partners into a broader **marketplace of tools for the community** to facilitate reuse and reproducibility. Additionally, we are already testing AI agents to boost accessibility and usability further with **“no code” interfaces** to further lower barriers for researchers to be able to analyze their own datasets, no matter how complex.

